# Evaluation of the Oxidative Stress Status in Zebrafish (*Danio rerio*) Liver Induced by Three Typical Organic UV Filters (BP-4, PABA and PBSA)

**DOI:** 10.3390/ijerph17020651

**Published:** 2020-01-19

**Authors:** Xinxin Huang, Yuanyuan Li, Tantan Wang, Hui Liu, Jiaqi Shi, Xuesheng Zhang

**Affiliations:** 1 School of Resources and Environmental Engineering, Anhui University, Hefei 230601, Anhui, China; xxhuangjy@163.com (X.H.); li__yyuan@163.com (Y.L.); wtt9698@163.com (T.W.); 2 College of Biological and Chemical Engineering, Jiaxing University, Jiaxing 314001, Zhejiang, China; leolau@163.com; 3 Nanjing Institute of Environmental Sciences of the Ministry of Environmental Protection, Nanjing 210042, Jiangsu, China; shijiaqi_jiayou@163.com

**Keywords:** organic UV filters, oxidative stress, lipid peroxidation, integrated biomarker response, zebrafish

## Abstract

Organic UV filters are a kind of emerging pollutants, which have been widely used in personal care products (PCPs). This study evaluated the effects of benzophenone-4 (BP-4), 4-aminobenzoic acid (PABA), and 2-phenylbenzimidazole-5-sulfonic acid (PBSA) on the selected indices of antioxidative responses in zebrafish (*Danio rerio*) liver. Zebrafish were exposed to two different doses (i.e., 0.5 and 5 mg L^−1^) of semi-static water with three individual compounds. Liver samples were collected on 7 and 14 days to analyze biochemical indicators, including superoxide dismutase (SOD), glutathione S-transferase (GST), reduced glutathione (GSH), and malondialdehyde (MDA). Oxidative stress occurred in zebrafish liver with significantly changed indicators during the whole exposure period. Different experimental groups could induce or inhibit the activity of antioxidant enzymes with varying degrees. With a prolonged exposure time and increased exposure dose, the hepatic lipid peroxidation was also obviously observed. Moreover, the toxicity order of three organic UV filters was analyzed using the integrated biomarker response (IBR) index and the results indicate that exposure to PABA for 7 days at 0.5 mg L^−1^ and PBSA for 7 days at 5 mg L^−1^ induced the most severe oxidative stress in the liver of zebrafish.

## 1. Introduction

In recent years, the amount of ultraviolet radiation has been increasing with the destruction of the ozone layer and its impact on human beings is well known. UV filters in both inorganic and organic forms can separately scatter or absorb UV-A (320–400 nm) and UV-B (280–320 nm) to protect hair and skin [1,2]. However, organic UV filters can be further metabolized in the body when they are absorbed via the skin and finally accumulated in the organism [3]. Organic UV filters are a kind of aromatic compounds widely used among personal care products (PCPs), such as benzophenone-3 (BP-3), benzophenone-4 (BP-4), 4-aminobenzoic acid (PABA), 4-methylbenzylidene camphor (4-MBC), 2-ethylhexyl 4-methoxycinnamate (EHMC), 2-phenylbenzimidazole-5-sulfonic acid (PBSA), octocrylene (OC) [4,5]. The levels of UV filters in cosmetics are generally from 0.1% to 10% [6]. At present, only 14 types of organic UV filters are allowed in cosmetics in the United States, and 26 of them are allowed in the European Union [5]. It has been reported that UV filters entered the aquatic system via either direct input of recreational activities (e.g., washing and swimming) or indirect input from the wastewater treatment plants (WWTPs) [7]. Kaiser et al. [6] observed that B-MDM, EHMC, and OCR were three main pollutants in sediments from the Rhine main area near Frankfurt (Hesse, Germany), with their highest levels of 62.2, 6.8, and 642 μg kg^−1^, respectively. Jurado et al. [8] reported the detection of a variety of benzophenones in groundwater of Barcelona and the maximum concentrations were measured as 36.6 ng L^−1^ (BP-4), 19.4 ng L^−1^ (BP-3), and 19.2 ng L^−1^ (BP-1). Balmer et al. [9] reported that the concentration of 4-MBC in fish from Swiss midland lakes can reach 166 ng g^−1^ on a lipid basis. Fent et al. [10] reported the chronic toxicity of organic UV filters, including 4-MBC, EHMC, BP-3, and BP-4, to *Daphnia magna*, but found the reduced reproduction and body length only at the highest concentration of 4-MBC (50 μg L^−1^).

Environmental contaminants can trigger the toxicity associated with oxidative stress. Oxygen toxicity is a harmful effect caused by cytotoxic reactive oxygen species (ROS), produced during metabolic transformation in organisms [11]. Under normal conditions, antioxidant defense system of organisms can remove ROS and protect complex biological macromolecules from ROS attack. However, when ROS levels induced by pollutants exceed the scavenging capability of antioxidant defense system, the balance will be destroyed, therefore weakening the activity of antioxidant enzymes. Organisms will suffer oxidative stress, resulting in lipid peroxidation (indicated by the significantly enhanced level of malondialdehyde (MDA)), chain breakage, enzyme-protein gluing, and even cell damage [12], death or canceration [13]. Antioxidant defense systems consist of a variety of enzymatic (e.g., catalase (CAT), superoxide dismutase (SOD), glutathione peroxidase (GPx), glutathione reductase (GR), and glutathione S-transferase (GST)) and non-enzymatic antioxidants such as reduced glutathione (GSH) [14,15].

Organic UV filters have been reported to be associated with the induction of oxidative stress in aquatic organisms. Gao et al. [2] reported that BP-3 at 1.0 μg L^−1^ could cause a significant increase of CAT activities and a significant reduction of GSH content of *Tetrahymena thermophile*. Campos et al. [16] confirmed that exposure to OC at 0.23 and 18.23 mg Kg^−1^ could lead to an increase in GST levels of *Chironomus riparius* while 4-MBC at 14.13 mg Kg^−1^ could cause reductions of CAT activity and an increase of GST activity in *Chironomus riparius*. Quintaneiro et al. [17] found that GST activity in zebrafish embryos (0–96 hpf) was elevated after exposure to 4-MBC at above 0.15 mg L^−1^. Owing to the complexity of organic UV filters in aquatic environment, the induction of oxidative stress with the significantly affected antioxidant defenses can be used to reflect the comprehensive pollution of PCPs in aquatic environment and to evaluate the environmental risks of polluted water.

Zebrafish (*Danio rerio*) have been widely used as indicator organism in toxicological studies of environmental pollutants [18,19]. The aim of this work was to (1) measure the levels of four oxidative stress indicators (i.e., SOD, GST, GSH, and MDA) in zebrafish liver exposed to these three pollutants, and (2) evaluate their toxicity order using the integrated biomarker response (IBR) index.

## 2. Materials and Methods

### 2.1. Reagents and Materials

The purity of three organic UV filters (i.e., BP-4, PABA, and PBSA, see their physico-chemical properties in Table 1) was 99%. Sodium chloride, acetic acid, and ethanol (95%) are of analytical grade and were purchased from Shanghai Aladdin Biochemical Technology Co., Ltd. (Aladdin, Shanghai, China). Enzymatic activity assay kits for SOD, GST, GSH and MDA and protein assay kits were purchased from Nanjing Jiancheng Institute of Bioengineering (Nanjing, China). Fish feed was purchased from a local aquarium store in Wuhan, China. Ultrapure water used throughout the whole experiment was produced via a Millipore Purification System (Millipore Elix 20, Millipore Corporation, Billerica, MA, USA).

The instruments used in the experiment included a high-speed refrigeration centrifuge (5810/5810R, Eppendorf, Hamburg, Germany), an electric homogenizer (F6/10, Jingxin Technology, Shanghai, China) and an ultraviolet-visible spectrophotometer (UV-1100, Shanghai Mespectra, Shanghai, China).

### 2.2. Fish Culture and Experimental Design

**Figure 1:**
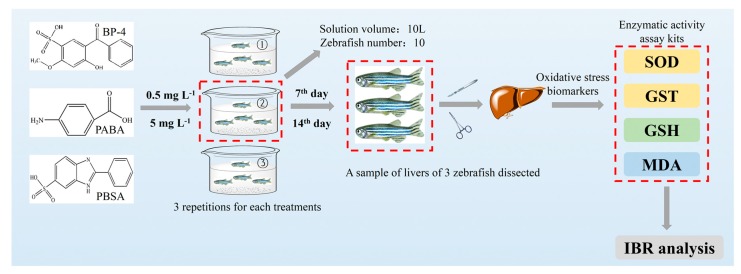
The testing procedures for oxidative stress evaluation of BP-4, PABA, and PBSA in zebrafish liver.

Experimentation was performed in accordance with the laboratory animal welfare of China and was approved by the animal ethics committee of Anhui University (No. 201916; effective date, 15 March 2019). The experiment was carried out according to the animal protection policy of Anhui University and an approved animal use agreement. The zebrafish (2.0 ± 1.0 cm and 0.2 ± 0.1 g) used in this study were purchased from the Institute of Hydrobiology of the Chinese Academy of Sciences (Wuhan, Hubei). Zebrafish were domesticated in freshwater over 72 h of aeration for a week. Fish were fed once a day according to 1% of the body weight. Only when the mortality rate is less than 1% during domestication can zebrafish be cultured in laboratory. Then, under natural light, zebrafish were cultured in the circulation system at 25 °C with pH 7.5 before further experiments. Semi-static mode was used for water change (every 24 h). Prior to normal experiments, the acute toxicity of three compounds was assessed via water exposure and the results suggest that the lethality of these compounds did not exceed 50%, even at the dose of 100 mg L^−1^. The doses of BP-4, PABA, and PBSA were selected at 0.5 mg L^−1^ and 5 mg L^−1^, which are higher than their environmental levels and the higher dose did not exceed 1/20 of LD_50_. At different exposure times, each ten zebrafish after domestication as an experimental group were exposed to 10 L experimental solutions containing 0, 0.5, or 5 mg L^−1^ of BP-4, PABA, and PBSA, respectively. Three replicates were performed for each experimental group. Fish were starved for 24 h prior to sampling to avoid prandial effects and to prevent the deposition of feces in the course of the assay. Livers of three zebrafish were individually taken out from 7 days and 14 days of exposure for determination of oxidative stress biomarkers (i.e., SOD, GST, GSH, and MDA). The water conditions were maintained constant during the whole exposure period (Temperature: 25 ± 1 °C; pH: 7.5 ± 0.1; Dissolved oxygen: 7.05 ± 0.43 mg CaCO_3_ L^−1^; Conductivity: 521 ± 9.56 μS cm^−1^; Hardness: 123 ± 3.42 mg L^−1^). During the whole exposure, the behaviors (i.e., lethargy, anorexia, erratic swimming, exophthalmia, corneal opacity, visible deformity, and hemorrhage at the operculum, pectoral, and ventral areas) of zebrafish were observed for judging the pathogenicity of fish. In short, the testing procedures for oxidative stress are illustrated in Figure 1.

### 2.3. Sample Preparation and Biochemical Analysis

The dissected liver was washed with 0.9% normal saline after zebrafish were frozen to death, then dried with filter paper and weighed. The homogenate produced by Ultra Turrax homogenizer in 0.9% normal saline was centrifuged at 8000 r/min for 10 min at 4 °C. The clear supernatant extract was analyzed for enzymatic activity.

All indicators were determined according to the manufacturer’s instructions. SOD activity was determined based on the principle that SOD could inhibit the reactivity of O_2_^−•^ [20]. GST activity was calculated by spectrophotometric determination of 1-chloro-2,4-dinitrobenzene (CDNB) and GSH at 412 nm [21]. MDA content was tested by method of thiobarbituric acid (TBA) [22]. Under acidic conditions (e.g., glacial acetic acid) and a high temperature (95 °C) for 40 min, the determination of MDA contents in zebrafish liver involved in the reaction between MDA and TBA to form the red complex that can be detected by a UV-VIS spectrophotometer at 523 nm. GSH levels were assayed using the method described by Jollow et al. [23]. The protein concentration measured by the Bradford method [24] was used to correct the activity of antioxidant enzymes.

### 2.4. Determination of the Concentrations of Three Compounds in Exposure Medium

The actual concentrations of BP-4, PABA, and PBSA in exposure water were determined using a HPLC system (Waters e2695). The mobile phases included methanol (80%) and 0.25% ethanoic acid in water (20%) with a flow rate of 1.0 mL/min. The detection wavelength was set as 310 nm for all three compounds. The column (Agilent ZORBAX SB-C_18_, 150 × 4.6 mm, 5 μm) temperature was 25 °C, and the injection volume was 10 μL. In addition, for preparation of the calibration curve, these compounds were dissolved in methanol at 100 mg L^−1^ as stock solutions. The stock solution was diluted to different concentrations (0.25, 1, 5, 10, and 50 mg L^−1^) in a 10 mL volumetric flask. The *R*^2^ values of the calibration curves for BP-4, PABA, and PBSA were 0.998, 0.996, and 0.995, respectively.

### 2.5. Integrated Biomarker Response (IBR)

Multiple biomarkers were combined into a general-purpose IBR index, by which the toxicity of three compounds can be directly reflected and proposed by Beliaeff et al. [25]. A brief calculation of IBR is given here. The formula for standardized data (Y) is as follows:

Y = (X − m)/S(1)

where X = the value of each biomarker responses, m = the mean value of the biomarker, and S = the standard deviation of the biomarker.

Z was computed through Z = Y in the case of activation or Z = −Y in the case of inhibition. The minimum (min) is provided by Y. Then, the score of a given biomarker (S) was obtained from S = Z + |min|, where S ≥ 0 and |min| is the absolute value.

Star plots can visually show the outcomes of biomarkers. The area of star plots (A_i_) was obtained from the following equation:

(2) $$\beta \;=\;\mathrm{Arctan}(\frac{S_{i+1}\sin\alpha }{S_{i}-S_{i+1}\cos\alpha }),$$

(3) $$A_{i}=\frac{S_{i}}{2}{\sin\beta (S}_{i}{\cos\beta +S}_{i}\sin\beta ),$$

where α = 2π/n radians; S_i_ = the obtained value of each biomarker, and S_n+1_ = S_1_.

When only four biomarkers are selected, the area formula was simplified to

(4) $$A_{i}{\;=\;S}_{i}S_{i+1}/2,$$

The value of IBR was calculated as

(5) $$\mathrm{IBR}=\sum_{n-1}^{i}A_{i},$$

where n = the number of biomarkers, which plays a key role [26].

### 2.6. Statistical Analysis

The SPSS statistical package program (ver. 22.0, IBM, Chicago, IL, USA) for Win 7.0 was used for a statistical analysis. Prior to a One-way ANOVA analysis, the standardization and homogeneity of data were checked by Kolmogorov–Smirnov test and Levene test, respectively. One-way ANOVA and Dunnett test were used to show difference between the control and the experimental treatments. Duncan’s multiple range test was conducted to identify significant difference among groups. Significant differences between groups could be illustrated by Duncan’s test. Significant difference was divided into two levels, i.e., significant (*p* < 0.05) and extremely significant (*p* < 0.01). All the results are represented as means ± S.D., and Origin 2017 (Origin Lab, OriginLab Corporation, Northampton, MA, USA) was used for figure plotting.

## 3. Results

In the present study, no pathogenic or dead fish were observed during the whole domestication and exposure (14 days). Indicators of oxidative stress included antioxidant enzymes (i.e., SOD and GST) and non-enzyme antioxidant (i.e., GSH and MDA). Changes of these indicators among different treatments are discussed in the next section. The actual levels of BP-4, PABA, and PBSA were monitored throughout the exposure duration (Table 2). The results indicate no significant difference (within 10%) between the nominal (i.e., 0.5 and 5 mg L^−1^) and measured concentrations. Hence, the nominal concentrations were used in the following discussions.

### 3.1. Antioxidant Enzyme Activities

**Figure 2:**
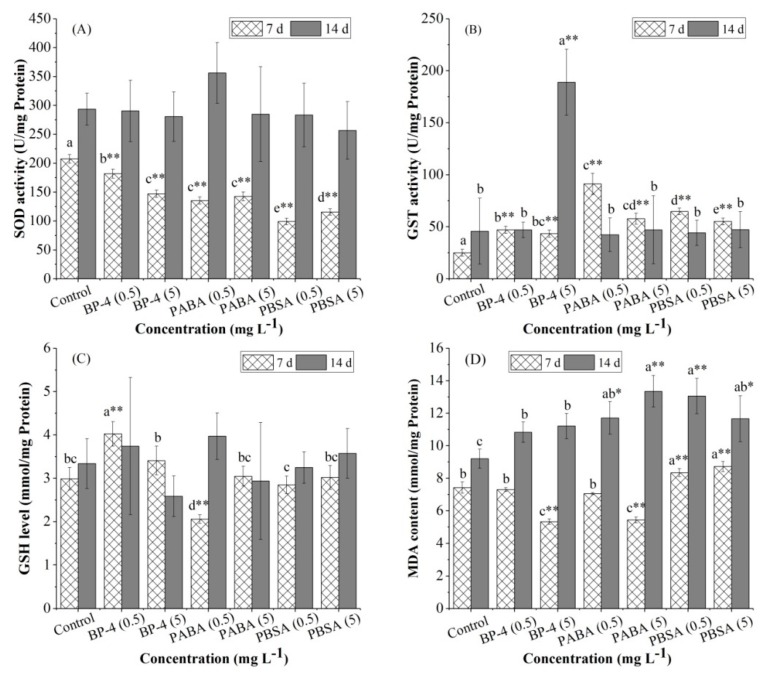
Effect of exposure to BP-4, PABA, or PBSA (0.5 and 5 mg L^−1^) for 7 and 14 days on the activity of biochemical indicators (**A**, SOD; **B**, GST; **C**, CSH; **D**, MDA) in zebrafish liver. Data are expressed as means ± SD, *n* = 3 for each data point. Superscript letters a–e indicate differences among the experimental treatments at the same exposure time. * Significantly different from controls (*p* < 0.05), ** Highly significantly different from controls (*p* < 0.01).

The effect of BP-4, PABA, and PBSA with two different concentrations on the activity of SOD and GST in zebrafish liver is shown in Figure 2. Compared with the control group after 7 days, SOD activity in all experimental groups decreased significantly (*p* < 0.01) (Figure 2A) and the decreases for the treatments by BP-4, PABA, and PBSA were 12.4%, 29.1%, 34.8% for 0.5 mg L^−1^ and 31.3%, 52.2%, and 44.4% for 5.0 mg L^−1^, respectively. After 14 days of exposure, no significant change (*p* > 0.05) in SOD activity was detected.

Contrarily to the trend of SOD activity, GST activity increased significantly in all experimental groups after 7 days of exposure (*p* < 0.01) (Figure 2B). With the extending exposure time, GST activity in all experimental groups, except the group of BP-4 (5 mg L^−1^), returned to the level of the control group. After 14 days of exposure, the group of BP-4 (5 mg L^−1^) showed a significant downward trend and the change of GST activity became the greatest.

### 3.2. GSH Levels

During the whole exposure, the change of GSH levels in zebrafish liver is illustrated in Figure 2C. After 7 days of exposure, GSH levels in low-dose groups (0.5 mg L^−1^) of BP-4 and PABA altered significantly (*p* < 0.01), but no significant variation (*p* > 0.05) was observed in other groups. GSH levels were significantly induced in the group of BP-4 (0.5 mg L^−1^), but significantly inhibited in the group of PABA (0.5 mg L^−1^) (*p* < 0.01). After 14 days of exposure, no significant change in GSH levels was observed among the experimental groups.

### 3.3. MDA Contents

Figure 2D exhibits the change of MDA contents. After 7 days of exposure, MDA contents were significantly decreased (*p* < 0.01) in the treatments, including BP-4 (5 mg L^−1^) and PABA (5 mg L^−1^), but all the treatments of PBSA (0.5 and 5 mg L^−1^) were significantly increased (*p* < 0.01). After 14 days of exposure, MDA contents were highly induced (*p* < 0.05 or *p* < 0.01) in all the treatments, particularly for PABA (5 mg L^−1^) and PBSA (0.5 mg L^−1^) (*p* < 0.01), except for the groups of BP-4 (0.5 and 5 mg L^−1^).

### 3.4. IBR Index

**Figure 3:**
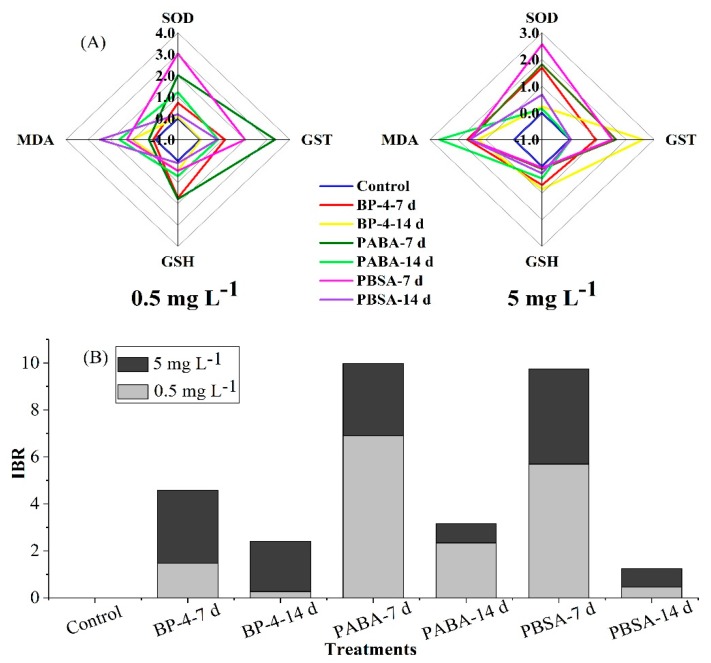
Biomarker star plots (**A**) and the calculated IBR values (**B**) of all biochemical parameters measured in zebrafish liver after exposure to BP-4, PABA, or PBSA (0.5 and 5 mg L^−1^) for 7 and 14 days.

In this study, the standardized values of four biomarkers determined on the 7th and 14th day of exposure are presented in Figure 3A, and the calculated IBR values are shown in Figure 3B. The IBR values for three chemicals-treated groups ranged from 1.24 for PBSA after 7 days (0.47 for 0.5 mg L^−1^ and 0.77 for 5 mg L^−1^) to 9.99 for PABA after 14 days (6.89 for 0.5 mg L^−1^ and 3.09 for 5 mg L^−1^). This indicates that at the test concentration of 0.5 mg L^−1^, zebrafish liver was most severely affected by oxidative damage in the following order: PABA-7 d > PBSA-7 d > PABA-14 d >BP-4-7 d > PBSA-14 d > BP-4-14 d > Control group. Comparatively, at the exposure level of 5 mg L^−1^, zebrafish liver was influenced by oxidative damage in the following order: PBSA-7 d > BP-4-7 d ≈ PABA-7 d > BP-4-14 d > PBSA-14 d ≈ PABA-14 d > Control group.

## 4. Discussion

It has been documented that organic UV filters could be released into the aquatic environment, accumulated by organisms in the food chain, therefore posing potential risks on fertility and reproduction of fish species [27,28]. According to the data obtained from this study, BP-4, PABA, and PBSA could cause oxidative damage to fish liver under different exposure doses and durations.

### 4.1. Antioxidative Responses

Measuring the effects of pollutants on organisms by the changes of key enzymes in specific reactions is a widely used method to study the status of oxidative stress [29]. SOD, as the first line of defense against oxidative stress, can catalyze the mutation of O_2_^−•^ and convert it into H_2_O and H_2_O_2_. GST, a phase II detoxification metabolic enzyme, can catalyze the binding of electrophilic groups of xenobiotics with sulfydryl groups of GSH to increase its hydrophobicity. GST also has GPx activity, which can inhibit lipid peroxidation [30]. In this experiment, compared with the control group, SOD activity decreased significantly in all the groups after 7 days of exposure to three chemicals. Excessive ROS production in fish after exposure, which exceeds the ability of SOD to remove ROS, was a cause of the results [31]. Similar results were also reported by Li et al. [32], in which SOD activity in the liver of *Carassius auratus* was inhibited after 14 days of exposure to highly fluorinated PFDDs (100 µmol kg^−1^). Falfushynska et al. [33] found that the gibel carp *Carassius auratus* gibelio inhabiting both upstream and downstream of the dam of Kasperivtci HPP in the West Ukraine showed a similar response in the decrease of SOD activity. Aytekin et al. [34] reported an inhibited SOD activity in the liver of *Oreochromis niloticusin* exposed to 0.6, 3.0, and 6.0 mg L^−1^ of Cu for 15 days. On the seventh day of exposure, GST activity increased more remarkably (*p* < 0.01) in all the experimental groups than those in controls, indicating that GST participated in the detoxification of these UV filters. Assessment of the effect of endosulfan in different concentration ranges on clams also showed a similar detoxification mechanism [30]. After 14 days of exposure, hepatic GST activity induced by BP-4 (5 mg L^−1^) increased significantly, which might be due to the accumulation of pollutants. These results indicate that GST was involved in the biotransformation of BP-4, and the complex of GST and BP-4 was produced to detoxify the toxicants in zebrafish liver.

GSH can detoxify not only by acting as a substrate of GPx and GST, but also by directly binding to ROS and electrophilic compounds [35,36]. Previous studies have clearly demonstrated that exposure to organic pollutants resulted in the increase or decrease of GSH levels in organisms depending on the exposure species, exposure duration and dose [37,38]. In this study, significant changes in GSH levels were found in the low-dose group after 7 days of exposure. The significantly increased GSH levels in the group of BP-4 (0.5 mg L^−1^) probably resulted from the enhanced hepatic uptake of amino acid substrates and the activities of biosynthetic enzymes in order to protect the organisms against oxidative damage [39]. The consumption of GSH due to the direct scavenging of ROS or as a co-factor for GST/GPx activities may significantly decrease GSH levels in the group of PABA (0.5 mg L^−1^) [40]. Moreover, the reaction of GSH during xenobiotic exposure might have been affected by the dose saturation phenomenon. In other words, GSH levels may not change significantly when the concentrations of test chemicals reach a certain dosage. This suggests that other detoxification systems may be involved in the reaction, or GSH is not very sensitive to those chemicals [41], or GSH contained in fish feed may have an enduring impact on the experiment during domestication [42]. In this study, the antioxidant responses were saturated with PABA group at 5 mg L^−1^ rather than 0.5 mg L^−1^. This suggests that GSH may be more sensitive to low doses and suitable for evaluating the antioxidant status of three organic UV filters at low levels.

### 4.2. Estimation of Lipid Peroxidation

Xenobiotics induce zebrafish to produce a large number of oxygen free radicals and will combine with unsaturated fatty acids in biofilm and cause lipid peroxidation [43]. MDA is a major degradation product of lipid hydroxides (LPO) and is often used as an effective biomarker for evaluating LPO when aquatic species are exposed to pollutants [44]. The level of MDA indirectly reflects the severity of free radical attack on body cells [45]. In the current study, MDA contents of PABA and PBSA at different concentrations increased significantly after 14 days of exposure. With the extension of the exposure time, the increased MDA content in zebrafish liver may indicate a significant time-dependent effect [46,47]. It can therefore be inferred that exposure of xenobiotics at high concentrations resulted in excessive generation of ROS in a short time. However, the ability of the antioxidant system to eliminate ROS was limited, while the remaining oxygen free radicals attacked the polyunsaturated fatty acids in biofilm, leading to the formation of lipid peroxides, i.e., lipid peroxidation, and also an increased MDA content [45,48]. Previous studies have also confirmed the lipid peroxidation in zebrafish liver exposed to triazophos, which was based on the significantly enhanced MDA content in the high-dose groups and a longer exposure time [47]. In contrast, the growth trend of MDA content in PBSA-treated groups was more obvious, indicating that PBSA was more likely to induce ROS production in fish liver, resulting in damage on the cell membrane.

Moreover, after 14 days of exposure to PABA and PBSA, the increase was only recorded in hepatic MDA contents. Similar results were also reported by Li et al. [49], in which they found that MDA contents in the liver of *Carassius auratus* were increased significantly after 2 days of exposure of 10 μg kg^−1^ 2,2′,4-Tris-CDPS, while no significant change in antioxidant enzymes was detected. It was thus speculated that no obvious relationship existed between MDA contents and other oxidative indicators (SOD, GST and GSH) after exposure of PABA and PBSA. Yonar et al. [50] reported that SOD activity, GST activity and MDA contents significantly increased after exposure of *Common carp* to chlorpyrifos (0.080 mg L^−1^) for 14 days, while CAT activity and GPx activity significantly decreased. An increase in MDA contents may indicate tissue damage caused by oxidative radicals, which might be reflected by other indicators (e.g., CAT and GPx activity). The findings of this study reinforce that MDA contents might be more suitable as the short-term toxicity index of PABA and PBSA. Moreover, no significant difference could occur in oxidative indicators (SOD, GST and GSH) after 14 days of exposure compared with the control group, depending on time and dose of application as well as the susceptibility of exposed species. However, as a final product of lipid peroxidation, MDA can increase continually by accumulating in fish tissues. MDA contents provide more direct evidence of the toxic process caused by free radicals than other indicators, which can be further confirmed by the determination of ROS levels.

In summary, exposure to organic UV filters can not only induce the increase of oxidative stress level in zebrafish liver, but also lead to lipid peroxidation. Antioxidants work together to remove ROS and protect the body from damage of free radicals [38].

### 4.3. Comparison of the Oxidative Stress-Inducing Potentials of Three Organic UV Filters in Zebrafish Liver

The toxicity difference of BP-4, PABA, and PBSA was analyzed and compared by the IBR index, which combines different biomarker signals to describe the “health status” of organisms. It is of great environmental significance to estimate the toxic effects of pollutants with specific enzymes and to use them as the biomarkers for early warning of water contamination [51]. Generally, the higher the IBR value is, the greater the environmental pressure will be. The IBR data in this study showed that PABA-7 d at 0.5 mg L^−1^ and PBSA-7d at 5 mg L^−1^ can cause the more severe damages in zebrafish liver. The IBR values decreased gradually with the prolonged exposure time, indicating that zebrafish liver could weaken the oxidative damage caused by these chemicals via its self-regulation mechanism. In the later stage of pollutant exposure, zebrafish liver could effectively eliminate the harmful free radicals produced during interactions of UV filters and zebrafish liver. In addition, it is worth noting that the stress of PABA on zebrafish liver in low-dose group is more serious than that of the other two compounds, which needs to be given great health concern.

### 4.4. Limitations of the Present Study

This study explored the effect of exposure to organic UV filters on hepatic antioxidant response in zebrafish. However, some limitations should also be considered. First, the doses of the three test compounds were higher than their environmental levels, and the dose-dependent effect was not observed due to the narrow concentration ranges. Secondly, though the present study demonstrates that organic UV filters (BP-4, PABA and PBSA) may affect the antioxidant system of zebrafish, the molecular mechanisms of oxidative stress need to be further clarified. Finally, determination of the concentrations of organic UV filters in zebrafish liver will facilitate understanding on the correlation of their bioaccumulation and hepatic antioxidant response.

## 5. Conclusions

In conclusion, the present study demonstrates that BP-4, PABA, and PBSA (0.5–5 mg L^−1^) could cause significant changes in SOD, GST, GSH, and MDA in zebrafish liver after exposure from 7 to 14 days. These phenomena indicate that exposure to these organic UV filters could increase ROS production and cause oxidative damage in fish liver. With the increase of exposure time and dose, MDA contents increased significantly, suggesting the presence of lipid peroxidation. The calculated IBR values suggested that exposure to PABA for 7 days at 0.5 mg L^−1^ and PBSA for 7 days at 5 mg L^−1^ had the most severe toxicity on the hepatic antioxidative defenses in zebrafish liver. In short, the data obtained in this study provide a scientific basis for the ecological risk assessment of different organic UV filters and their toxicological mechanisms are the future research direction needing more attention.

## Figures and Tables

**Table 1:** Physico-chemical properties of three tested chemicals.

Compounds	Chemical Structure	Log *K*_ow_^a^	Solubility (g L^−1^) ^b^
BP-4	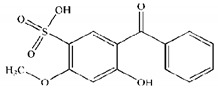	0.37	250
PABA	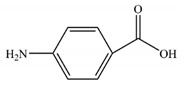	0.83	6.11
PBSA	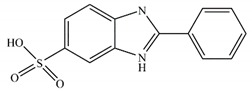	−0.16	23.6

^a^ Octanol/water partition coefficient, data from reference [2]. ^b^ Data calculated in ECOSAR (Ecosar Application 2.0, US Environmental Protection Agency, Washington, DC, USA).

**Table 2:** Nominal and corresponding measured concentrations of the three compounds.

Compounds	Nominal Concentration	Measured Concentration
	(mg L^−1^)	(mg L^−1^) ^a^
BP-4	0.5	0.47 ± 0.03
	5	4.58 ± 0.23
PABA	0.5	0.46 ± 0.04
	5	4.79 ± 0.66
PBSA	0.5	0.53 ± 0.06
	5	5.38 ± 0.83

^a^ Data are presented as mean ± S.D. and determined using HPLC.
